# Molecular Signature of Monocytes Shaped by the *Shigella sonnei* 1790-Generalized Modules for Membrane Antigens Vaccine

**DOI:** 10.3390/ijms25021116

**Published:** 2024-01-17

**Authors:** Serena Tondi, Emilio Siena, Ahmed Essaghir, Benoît Bozzetti, Viviane Bechtold, Aline Scaillet, Bruna Clemente, Mariateresa Marrocco, Chiara Sammicheli, Simona Tavarini, Francesca Micoli, Davide Oldrini, Alfredo Pezzicoli, Martina Di Fede, Michela Brazzoli, Cristina Ulivieri, Francesca Schiavetti

**Affiliations:** 1 Preclinical Research & Development, GSK, 53100 Siena, Italy; serena.tondi@ircc.it (S.T.);; 2 Department of Life Sciences, University of Siena, 53100 Siena, Italy; 3 Preclinical Research & Development, GSK, 1330 Rixensart, Belgium; 4 GSK Vaccines Institute for Global Health S.R.L. (GVGH), 53100 Siena, Italy

**Keywords:** Generalized Modules for Membrane Antigens (GMMA), *Shigella sonnei*, vaccine, immunity, monocytes, transcriptomics

## Abstract

Shigellosis, an acute gastroenteritis infection caused by *Shigella* species, remains a public health burden in developing countries. Recently, many outbreaks due to *Shigella sonnei* multidrug-resistant strains have been reported in high-income countries, and the lack of an effective vaccine represents a major hurdle to counteract this bacterial pathogen. Vaccine candidates against *Shigella sonnei* are under clinical development, including a Generalized Modules for Membrane Antigens (GMMA)-based vaccine. The mechanisms by which GMMA-based vaccines interact and activate human immune cells remain elusive. Our previous study provided the first evidence that both adaptive and innate immune cells are targeted and functionally shaped by the GMMA-based vaccine. Here, flow cytometry and confocal microscopy analysis allowed us to identify monocytes as the main target population interacting with the *S. sonnei* 1790-GMMA vaccine on human peripheral blood. In addition, transcriptomic analysis of this cell population revealed a molecular signature induced by 1790-GMMA mostly correlated with the inflammatory response and cytokine-induced processes. This also impacts the expression of genes associated with macrophages’ differentiation and T cell regulation, suggesting a dual function for this vaccine platform both as an antigen carrier and as a regulator of immune cell activation and differentiation.

## 1. Introduction

*Shigella* species (spp.) are Gram-negative, facultative anaerobic, and non-motile bacteria. Based on the structure of the lipopolysaccharide (LPS), it is possible to distinguish different serotypes [1,2,3]. *Shigella* spp. can invade the intestinal epithelium, causing bacillary dysentery, known as shigellosis, and *Shigella* spp.-derived infections are the leading cause of bacterial diarrhea worldwide, which is often correlated with a very high mortality rate [4]. The persistence of *Shigella* spp. within the intestinal mucosa triggers the activation of the innate immune system through bacterial components, with LPS considered the most crucial one. LPS is a molecule composed of hydrophobic lipid A and a hydrophilic polysaccharide called O-antigen (O-Ag) [5]. Recent findings indicate that bacteriophage-encoded glucosylation of *Shigella* O-Ag effectively shortens the LPS molecule, thereby facilitating bacterial invasion and enabling evasion of the innate immune response [6]. The innate immune response to *Shigella* spp. infection is characterized by the onset of acute inflammation, and in human subjects, the analysis of cytokine expression during the acute phase of the disease reveals an increase in pro-inflammatory genes, including IL-6, TNF-α, IL-8, TNF-β, and IL-1β [7,8].

Although shigellosis can be treated with antibiotics, the appearance of antibiotic-resistant strains of *Shigella* spp. secondary to drug overuse in human medicine and in the food industry remains one of the biggest problems in global health [9,10]. Multidrug-resistant outbreaks have recently been reported in high-income countries, as well [11].

Outer Membrane Vesicles (OMVs), which are naturally released by Gram-negative bacteria, are associated with many of their biological functions [12,13,14,15,16], and they have attracted attention in the vaccine development area [17]. Indeed, OMVs are composed of the constituents of the bacterial outer membrane (lipids, outer membrane proteins, and soluble periplasmic components), including antigens in their native environment, which can trigger an immune response without causing infection [18,19,20]. OMVs combine multiple antigen presentations to self-adjuvanticity and are easily uptaken by the cells of the immune system [12,13,14,15,16]. Nevertheless, the major limitations of their use are related to the presence of LPS, which is responsible for their high reactogenicity [21,22], and to the low production yields. To overcome these limitations, bacteria have been genetically mutated to increase vesicles’ blebbing and to reduce their reactogenicity, thus producing mutated OMVs called Generalized Modules for Membrane Antigens (GMMA) [23,24]. A first-generation GMMA-based vaccine was proposed against *S. sonnei* only [25]. Wild-type bacteria were modified by deleting *tolR* to increase vesicle blebbing and by deleting the *htrb* gene to obtain a penta-acetylated structure of lipid A with a reduced endotoxicity (1790-GMMA) compared to GMMA with hexa-acylated wild-type lipid A structure (wild-type GMMA) [26,27,28]. This *S. sonnei* GMMA vaccine candidate has been shown to elicit bactericidal anti-OAg antibodies when tested in clinical trials [25], and, more recently, a four-component OAg-based vaccine, including *S. sonnei*, *S. flexneri* 1b, 2a, and 3a GMMA, called altSonflex1-2-3, has been developed with the aim of protecting against the most prevalent *Shigella* serotypes [29,30]. Besides the ability of *S. sonnei* GMMA vaccine candidates to elicit bactericidal anti-OAg antibodies, their mode of action (MoA) at the cellular level is sparsely addressed. Previous in vitro studies conducted on human peripheral blood mononuclear cells (hPBMCs) demonstrated that lipid A mutation reduced the TLR4-dependent activation pathway and the release of pro-inflammatory cytokines [26] but still maintained the ability to activate monocytes, myeloid dendritic cells (mDCs), plasmacytoid dendritic cells (pDCs), B cells, NK cells, and γδ T cells [31]. Whether all of these immune cells are activated by a direct interaction with GMMA or become activated through a bystander effect remains unknown.

In this study, we have explored the interplay between *S. sonnei* GMMA and single immune cell populations. Through this investigation, we identified monocytes as the primary immune cell population in the peripheral blood involved in GMMA interaction. Moreover, by conducting an analysis of the GMMA-induced transcriptome in monocytes, we found a specific molecular fingerprint that distinguishes 1790-GMMA from their wild-type counterpart in terms of Differentially Expressed Genes (DEGs). This unique signature is primarily associated with inflammatory responses, including IFN responses, processes induced by IL1β/TNFα/IL15, and inflammasome activation. Furthermore, our findings suggest that the 1790-GMMA vaccine candidate has an influence on the expression of genes linked to antigen-presenting cell differentiation and the regulation of T cells.

This work contributes to unraveling the mechanisms through which 1790-GMMA operate and that support the dual role of this vaccine platform as an antigen carrier and a regulator of immune cell differentiation.

## 2. Results

### 2.1. S. sonnei 1790-GMMA Directly Interact with and Activate Monocytes, Thus Promoting the Production of Pro-Inflammatory Cytokines

We have previously demonstrated that *S. sonnei* 1790-GMMA are able to promote the release of pro-inflammatory cytokines from in vitro stimulated hPBMCs, such as IFN-γ, IL-12p70, IL-1β, IL-6, IL-8, and TNF-α [26]. The cytokine profile induced is similar in terms of quality but to a lesser extent compared with the wild-type counterpart. Interestingly, we also documented a 1790-GMMA-dependent activation of monocytes, dendritic cells, B cells, NK cells, and γδ T cells within hPBMCs [31]. Because single cell population among hPBMCs may be activated either through direct interaction with GMMA or through a bystander effect due to the nearby activated cells, we decided to identify the directly targeted cell population by using fluorescent 1790-GMMA. hPBMCs were treated with 1790-GMMA labeled with Alexa Fluor 488 (AF488), and the impact of AF488-positive cells measured through flow cytometry on monocytes, dendritic cells, B cells, T cells, and γδ T cells.

**Figure 1:**
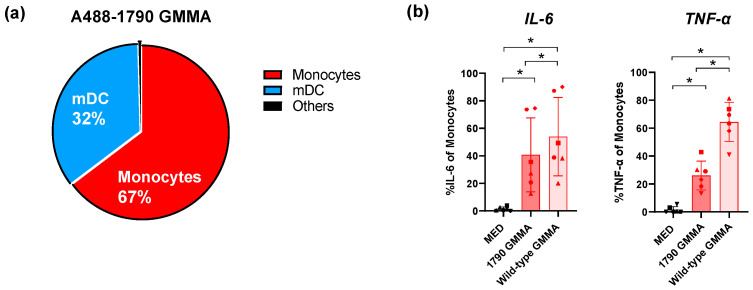
(**a**) Pie chart showing the *S. sonnei* AF488-1790-GMMA-positive cells after 1 h of stimulation. Monocytes (red) and mDCs (blue) have the highest percentages of cells positive for AF488-1790-GMMA, while the remaining 1% is represented by other immune cell populations (black). (**b**) Intracellular cytokine production by monocytes after 22 h of stimulation with 1790-GMMA (dark red) and wild-type GMMA (light red) in the presence of Brefeldin A. Histograms show the percentage of monocyte-producing IL-6 (left) and TNF-α (right). MED refers to negative control samples (medium treated). Percentages were calculated based on a manually gated cell population. Data are presented as mean value ± standard deviations (SDs) (*n* = 6 healthy donors). Statistical significance was estimated using the paired Wilcoxon test (* *p* < 0.05). Different symbols are referred to different donors tested.

The results revealed that monocytes and mDCs are the two main cell populations directly targeted by 1790-GMMA, thus representing 32% and 67%, respectively, of all GMMA-interacting cells. It is of note that 1790-GMMA were also found to directly interact with other immune cells, even if with a lower frequency of 1% (Figure 1a). Additionally, the comparison between 1790-GMMA and wild-type GMMA revealed that the induced modification of lipid A does not significantly affect the binding ability of 1790-GMMA to monocytes and mDCs (Supplementary Figure S1a). 

Intracellular cytokine staining performed on hPBMCs treated with 1790-GMMA in the presence of Brefeldin A, an inhibitor of intracellular protein transport and cytokine release used here to prevent cells’ activation via the bystander effect, further showed that 1790-GMMA induced a significant increase in the production of IL-6 and TNF-α in monocytes (Figure 1b) and mDCs (Supplementary Figure S1b) compared with unstimulated cells, although at a lower level than the wild-type GMMA, which was used as a positive control in this assay.

### 2.2. S. sonnei 1790-GMMA Are Internalized by Monocytes

Previous studies have suggested that OMVs, which are naturally secreted by all bacteria, shape the function of target cells by entering the host cells through different mechanisms, including endocytosis and membrane fusion [32]. Whether GMMA can be internalized by monocytes or remain on their surface is unknown. Therefore, we performed confocal microscopy analysis on monocytes treated with Alexa Fluor-647 *S. sonnei* GMMA (AF647), including both wild-type and 1790-GMMA, followed by an incubation with a primary antibody anti-*S. sonnei* GMMA and a secondary antibody conjugated with Alexa Fluor-546 (AF546), which enabled us to discriminate between GMMA attached on the cell surface (AF546 positive) from all of the GMMA interacting with monocytes (AF647 positive).

**Figure 2:**
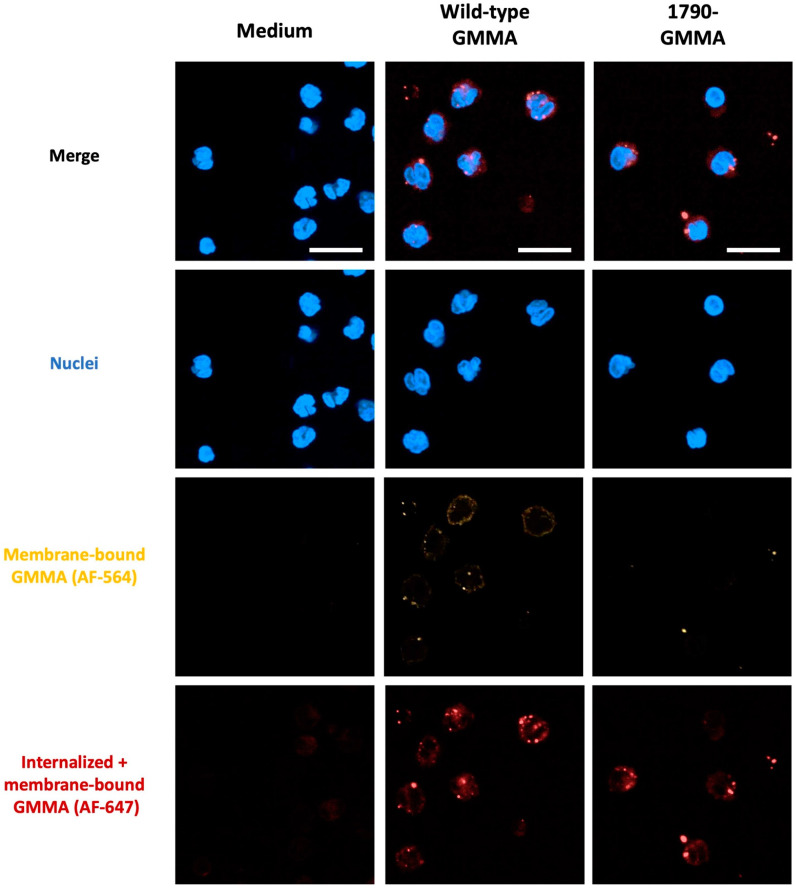
Confocal microscopy analysis of human monocytes treated for 1 h with wild-type GMMA or 1790-GMMA. Cell nuclei stained with DAPI (blue signal), plasma-membrane-bound GMMA (orange signal, AlexaFluor-546), and total (internalized + plasma-membrane-bound) GMMA (red signal, AlexaFluor-647) are shown. Untreated monocytes are also shown (medium). Scale bars 50 µm.

The results presented in Figure 2 demonstrate that both wild-type GMMA and 1790-GMMA are internalized by monocytes within as little as 1 h following treatment and provide the first evidence that GMMA internalization occurs independently of LPS lipid A’s structure.

### 2.3. 1790-GMMA and Wild-Type GMMA Induced a Distinct Transcriptional Program in Monocytes

Our data show that the 1790-GMMA candidate vaccine retains the ability to directly target and stimulate monocytes when compared with wild-type GMMA. In addition, a proteomic analysis revealed a similar composition between the two GMMA [30], suggesting that, by carrying the same protein cargo, both 1790-GMMA and wild-type GMMA might shape the function of this cell population in the same manner. Because our data demonstrate that both GMMA are internalized by monocytes, we asked whether the vaccine candidate 1790-GMMA and its wild-type counterpart induce a similar transcriptional program to deepen our knowledge of the impact of 1790-GMMA on monocyte function. 

**Figure 3:**
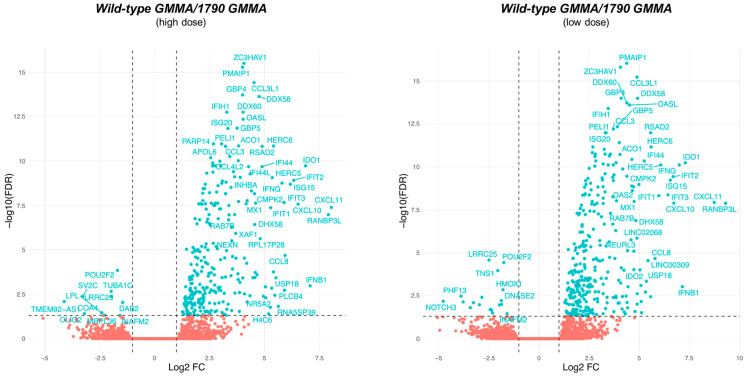
Volcano plots representing the comparison of the transcriptome modulation induced in monocytes upon stimulation with wild-type GMMA versus 1790-GMMA at a higher concentration (0.1 μg/mL), left plot, and a lower concentration (0.01 μg/mL), right plot. X axes represent the log2 scaled differential expression compared with the control group, while Y axes represent the statistical significance of the modulation (−log10 scaled Benjamini–Hochberg false discovery rate (FDR)). Genes with an FDR ≤ 0.05 and |log (FC)| ≥ 1 were assumed to be significantly modulated. Modulated genes are shown as blue dots upregulated on the right side and downregulated on the left side, while unregulated genes are shown as red dots. The vertical dotted lines indicate the +1 and −1 log2 fold change, while the horizontal dotted line indicates the 5% false discovery rate.

To this end, monocytes were treated with wild-type GMMA or with 1790-GMMA for 3 h at two different concentrations: a higher dose of 0.1 μg/mL (H) and a lower dose of 0.01 μg/mL (L). Differential gene expression analysis revealed that the treatment of monocytes with the higher dose of 1790-GMMA resulted in the significant modulation of 645 genes, whereas wild-type GMMA treatment regulated 1636 genes. A similar trend was observed at a lower dose, as 1790-GMMA induced the regulation of 480 genes, while wild-type GMMA regulated 1598 genes (Supplementary Figure S2). Given the differences in the magnitude of transcriptome modulation induced by 1790 and wild-type GMMA, a head-to-head comparison was performed to identify those genes that are differentially modulated between the two. For both high and low GMMA doses, the wild-type construct was found to induce a stronger transcriptome response (Figure 3). Interestingly, the number of differentially expressed genes identified across the high-dose and the low-dose comparisons was remarkably similar (high-dose comparison: 343, low-dose comparison: 385), and the pools of upregulated genes were mostly overlapping (Figure 3).

### 2.4. A Modular Framework Analysis Revealed the Transcriptional Fingerprinting of 1790-GMMA 

**Figure 4:**
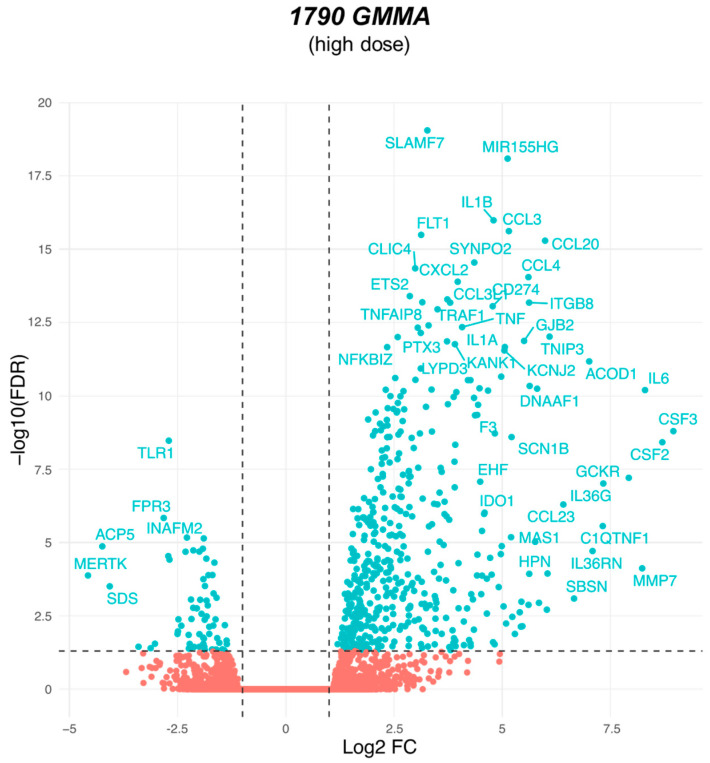
Volcano plot representing the transcriptome modulation induced in monocytes upon stimulation with 1790-GMMA at a higher concentration (0.1 μg/mL) compared with untreated monocytes. X axes represent the log2 scaled differential expression compared with the control group, while Y axes represent the statistical significance of the modulation (−log10 scaled Benjamini–Hochberg false discovery rate (FDR)). Genes with an FDR ≤ 0.05 and |log2 (FC)| ≥ 1 were assumed to be significantly modulated. The applied thresholds are represented by dotted lines, while modulated genes are shown as blue dots. Red dots represent unmodulated genes. The vertical dotted lines indicate the +1 and −1 log2 fold change, while the horizontal dotted line indicates the 5% false discovery rate.

Given that 1790-GMMA represent the vaccine candidate, we focused our analysis on the comparison between 1790-GMMA-treated and untreated monocytes. Overall, 645 genes were differentially expressed by monocytes upon 1790-GMMA treatment compared with untreated monocytes (Figure 4). 

**Figure 5:**
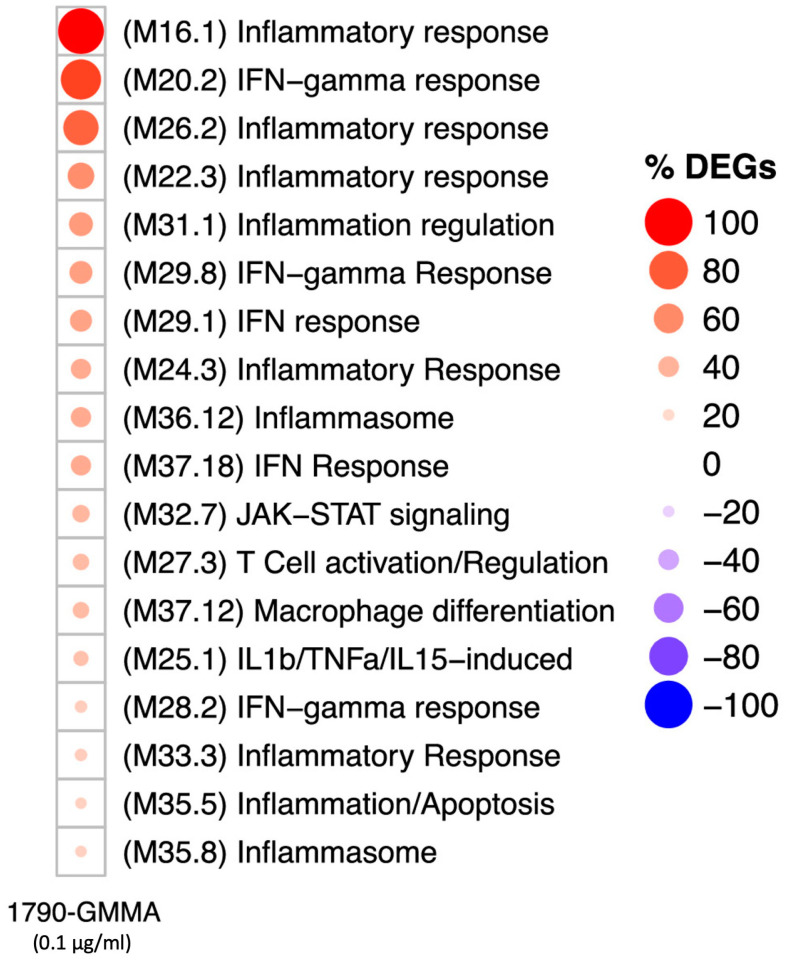
Monocyte signature induced by *S. sonnei* 1790-GMMA. Induction of 18 modules in monocytes after 3 h of stimulation with 1790-GMMA (average of six donors) at 0.1 μg/mL (higher dose). The circle size is proportional to the percentage of genes that are either up- or downregulated, while the circle color represents the direction of the modulation (red = % upregulated genes; blue = % downregulated genes). “DEGs” stands for Differentially Expressed Genes.

To facilitate the biological interpretation of gene expression data, we ran a functional enrichment analysis based on the blood transcriptional modules (BTMs) originally proposed by Bancherau et al. [33]. This approach was adopted to simultaneously reduce the amount of multiple testing and to facilitate biological interpretation. After mapping the assessed genes and filtering for those BTMs containing ≥ 25% modulated genes, we obtained 18 BTMs that represent the transcriptional fingerprint of 1790-GMMA-treated monocytes (Figure 5). 

**Figure 6:**
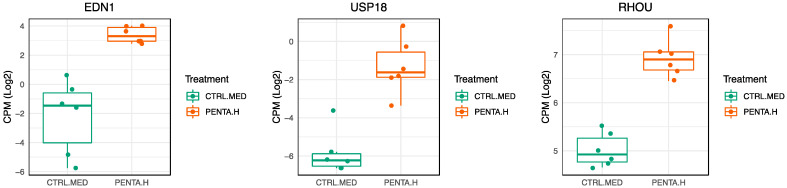
Comparison of DEGs belonging to the M37.12 module in monocytes treated with *S. sonnei* 1790-GMMA (0.1 μg/mL) or left untreated. Boxplots show the regulation levels of EDN1, USP8 and RHOU expressed as count per million reads mapped (CPM) in log2 in 1790-GMMA treated monocyte (orange) or untreated green (CTRL.MED) (*n* = 6 healthy donors).

The modules within the grid predominantly show associations with various biological processes, including the inflammatory response (M16.1, M26.2, M22.3, M24.3, M33.3), IFN-γ and IFN response (M20.2, M29.8, M29.1, M37.18, M28.2), IL1β/TNFα/IL15-induced processes (M25.1), inflammation, and inflammasome (M36.12, M35.5, M35.8). These findings are consistent with our previous findings [31], because a closer analysis of DEGs within these modules points towards the upregulation of regulatory pathways involved in the production of cytokines and chemokines (such as IL1β, IL6, TNF, IL1α, CCL2, and CCL3) as well as the regulation of genes involved in cellular activation (e.g., CD40). Notably, our analysis revealed the capacity of the 1790-GMMA to regulate the expression of genes associated with additional modules, such as macrophage differentiation (M37.12) and T cell activation/regulation (M27.3). In particular, in the M37.12 module, we found EDN1, USP18, and RHOU genes were upregulated by 1790-GMMA compared with untreated monocytes (Figure 6). Macrophages, together with DCs, are specialized antigen-presenting cells (APCs) that effectively capture, process, and present antigens, including those contained in vaccine formulations [34]. In this context, the new evidence that 1790-GMMA impact the differentiation program of monocytes into macrophages might represent additional information about the GMMA-based vaccines’ mode of action.

The EDN1 gene encodes for Endothelin-1, which promotes pro-inflammatory macrophages’ activation and macrophages’ differentiation into the M1 phenotype, which is characterized by the secretion of iNOS, IL-6, TNF-α, and IL-1α [35,36]. USP18 encodes for ubiquitin-specific peptidase 18, which has been shown to positively regulate the differentiation of CD11b dendritic cells, thereby promoting antigen presentation [37,38]. The RHOU gene, which encodes for a protein of the Rho family of GTPase, is implicated in several key macrophage functions, such as cell migration, phagocytosis, tissue remodeling, and the inflammatory response, through the activation of the nuclear factor kappa B (NF-κB), the master transcription factors of pro-inflammatory cytokines genes [39].

Overall, these findings suggest that 1790-GMMA, by increasing the expression of these genes, have the potential to stimulate the differentiation of monocytes into macrophages, thereby improving antigen presentation [40], and promote their migration. Importantly, by inducing the production of cytokines, such as IL-1, TNF-α, and interferons, upon pathogen recognition by macrophages, 1790-GMMA are likely to enhance the clearance of pathogens. Indeed, these cytokines have multiple effects, including enhancing inflammation, activating other immune cells, and promoting the recruitment of additional immune cells to the site of infection. 

## 3. Discussion

Despite continued scientific progress, the increase in the number of antimicrobial-resistant (AMR) pathogens and their spread remain some of the major challenges in the context of global health [9,10]. In this scenario, there is growing concern regarding antibiotic resistance in *Shigella* spp. [41,42,43], and the World Health Organization’s Global Antimicrobial Resistance Surveillance System has designated *Shigella* spp. as a priority pathogen warranting the development of new interventions [44].

GMMA-based vaccines, which are composed of outer membrane vesicles released by genetically modified bacteria, offer numerous advantages in comparison to classical vaccines. One significant benefit is their ability to carry multiple bacteria surface antigens; together with simplicity of manufacturing, these benefits support the possibility of formulating a candidate vaccine containing a mixture of vesicles released from different serotypes of the same bacteria. This results in broader protection against various strains and variants of the target pathogen [24,45]. This characteristic is of relevance for the development of a vaccine against *Shigella* spp. Another advantage lies in the intrinsic adjuvant properties of GMMA vaccines, as they can stimulate a stronger immune response without requiring additional adjuvants [24]. This can happen because of their membrane structure, which contains components like LPS and lipoproteins that, besides other bacteria-specific membrane proteins, trigger cytokine production, enhance antigen presentation, and ultimately boost both innate and adaptive immune responses [26,27,46]. The *S. sonnei* GMMA-based vaccine, which is composed of vesicles carrying a modified lipid A structure, has been shown to elicit both anti-LPS and anti-protein antibodies, with the former playing the most important function in the generation of bactericidal activity against *S. sonnei* [47,48]. 

The hypothesis that *S. sonnei* GMMA containing modified LPS (1790-GMMA) are likely to orchestrate the immune response by activating various immune cell populations was reinforced by our previous findings, which showed that this candidate vaccine activates APCs (monocytes and dendritic cells) and B and γδ T lymphocytes and promotes the production of pro-inflammatory cytokines, including IFNα, IL-10, IL-12p70, IL-1α, IL-6, TNFα, and IL-8, albeit in lower quantities compared to the GMMA variant with the wild-type lipid A [31]. Additionally, an increase in levels of IL-2, IL-4, Eotaxin, MCP-1, MIP-1α, MIP-1β, GM-CSF, IL-12p40, and IL-1β was detected [31]. 

While previous work has demonstrated that the candidate vaccine activates several immune cells in vitro [26,31], thus fostering knowledge of the mechanisms by which GMMA-based vaccines elicit protective immunity, experiments were carried out on hPBMCs, and no information was available on the specific targeted population. This information is crucial for deepening our comprehension of how GMMA interact with target cells and whether they elicit a particular molecular pattern to support the development of improved vaccines [23,24].

In this work, the data revealed that the main population interacting directly with *S. sonnei* GMMA consisted of monocytes (65%), followed by mDCs (34%), and other immune cell populations represent only 1%. In addition, by stimulating hPBMCs using both GMMA (1790-GMMA and wild-type GMMA) and treating cells with an intracellular trafficking inhibitor to avoid cytokine secretion and thus blocking a potential bystander effect (Brefeldin A), we demonstrated that monocytes produce pro-inflammatory cytokines, such as IL-6 and TNFα, through direct GMMA activation. In agreement with the results previously obtained by measuring the amount of secreted cytokines by hPBMCs [31], we showed that 1790-GMMA induced a lower level of cytokine production with respect to wild-type GMMA, thus confirming that the lipid A modification attenuates the activation induced by GMMA but does not reduce it completely. This also suggests that the residual cellular activation and cytokine production could be due to other immune receptors in addition to Toll-Like Receptors (TLRs) [26].

We demonstrated that 1790-GMMA not only engaged directly with monocytes but also underwent internalization within 1 h following stimulation; notably, this internalization occurred independently of the LPS structure. This interaction and subsequent internalization process holds potential significance in terms of triggering the immune response. Monocyte internalization of vaccine components improves their capacity for antigen presentation, potentially facilitating the activation of T cells, as suggested by the observed regulation of genes associated with T cells’ activation/regulation module (M27.3). This could result in a swifter and more robust immune response upon subsequent encounters with the [49]. 

In addition, data about the percentage of DEGs in GMMA-treated monocytes compared with untreated cells indicated that both the wild type and 1790-GMMA induce a certain level of transcriptomic regulation, thus up- or downregulating different genes. In particular, 1790-GMMA induce the differential expression of a lower number of genes than wild-type GMMA but maintain the ability to activate monocytes despite having a modified lipid A. Indeed, we found upregulation in the expression of genes related to cytokine and chemokine production (IL1B, IL6, TNF, IL1A, CCL3, and CCL4) as well as regulation of genes involved in cellular activation (CD40), which is in agreement with our previous studies [31]. 

Afterward, an unbiased analysis framework was employed to facilitate the biological interpretation of gene expression data. Related transcripts were grouped into sets of co-regulated genes known as blood cell transcriptional modules (BTMs). The data obtained from this additional analysis highlighted that the modules in which the percentage of DEGs was higher after 1790-GMMA treatment were predominantly related to inflammatory response, IFN-gamma and IFN response, IL1b/TNFa/IL15-induced processes, inflammation, and inflammasome. It is of note that the modular analysis unveiled the capacity of 1790-GMMA to exert control on genes associated with less expected modules, such as macrophage differentiation and T cell activation/regulation modules. This finding offers compelling proof of a robust activation of the immune system induced by GMMA, suggesting that GMMA can stimulate the differentiation of monocytes into macrophages, as evidenced by the increased expression of END1, UPS18, and the RHOU gene, which are associated with the activation of macrophages, particularly in terms of their capacity for cytokine production and enhancement of antigen presentation abilities [35,36,37,38].

These data therefore suggest a possible impact of 1790-GMMA on adaptive immunity, because macrophages possess the capability to process and display antigens derived from engulfed pathogens and vaccines to T cells. This step is critical in initiating the adaptive immune response, encompassing the activation of cytotoxic T cells and the generation of antibodies by B cells [50,51]. Furthermore, macrophages assume a role in immune regulation through phagocytosis and the clearance of dead cells and other debris, which aims at maintaining the immune balance (homeostasis) and the prevention of excessive immune reactions [52]. Another significant aspect to consider is the engagement of dendritic cells, the most efficient APCs, which our data demonstrated to represent the second target population directly interacting with 1790-GMMA.

Understanding the MoA of a vaccine candidate can provide critical information that will help with the rational design of new-generation vaccines. In this work, a much more in-depth study of 1790-GMMA has been conducted. The results show that 1790-GMMA directly interact with monocytes, and, as consequence of this interaction, vesicles are internalized, thus inducing transcriptional changes. Indeed, the data generated at the gene level suggest that 1790-GMMA, by activating monocytes, may play a role in orchestrating the immune response after vaccination by inducing macrophage differentiation, which will indirectly impact the adaptive response. 

Nevertheless, additional research will be essential to confirm the gene expression data at the cellular level and to explore how GMMA-induced activation of innate immunity influences the subsequent adaptive response to antigens. Such investigations will not only yield further insights into the underlying mechanisms, but also potentially offer guidance for refining immunization strategies to enhance GMMA effectiveness.

## 4. Materials and Methods

### 4.1. Isolation of hPBMCs and Monocytes from Healthy Donors

Cryopreserved hPBMCs were isolated from healthy individuals at Empoli Hospital (Italy) and Tivoli Hospital (Belgium). This research was conducted with the approval of local ethics committees and adhered to good clinical practice, following the principles outlined in the 1975 Declaration of Helsinki. Monocytes were isolated using an immunomagnetic negative selection method, as per the manufacturer’s instructions, utilizing the EasySep human monocyte isolation kit from STEMCELLS Technologies (Vancouver, BC, Canada).

### 4.2. Thawing of hPBMCs

Thawing of cryopreserved hPBMCs (derived from healthy individuals at Empoli Hospital (Empoli, Italy) and Tivoli Hospital (Tivoli, Italy)) was carried out at 37 °C, followed by two washes with a pre-warmed solution (consisting of PBS without Ca^+^ and Mg^+^ from Gibco Life Sciences (Carlsbad, CA, USA), 2.5 mM of EDTA from Euroclone (Pero, Italy), and 20 μg/mL of DNAse from Boheringer Mannheim (Mannheim, Germany)) through centrifugation at 1200 rpm for 10 min. Subsequently, hPBMCs were suspended in complete RPMI-1640 medium, which included 1% non-essential amino acids and 1% sodium pyruvate from Gibco Life Sciences, 1% Penicillin/Streptomycin/Glutamine from Euroclone, and 10% heat-inactivated fetal bovine serum (FBS) from Hyclone (Logan, UT, USA). To assess cell viability, a Trypan blue dye exclusion assay was performed using the Vi-Cell-XR instrument from Beckman Coulter (Brea, CA, USA). The viability of the thawed hPBMCs ranged between 90% and 98% of the total cell population.

### 4.3. Labeled S. sonnei GMMA Preparation 

*S. sonnei* GMMA wild-type and 1790-GMMA were concentrated to 10 mg/mL. Alexa Fluor-488 succinimidyl ester (Thermo Fisher A20000, Waltham, MA USA) and Alexa Fluor-647 succinimidyl ester (Thermo Fisher A20006) were directly added in a 20:1 Dye:GMMA *w*/*w* ratio and left for 1h at RT in a dark room to favor chemical conjugation on primary amines (R-NH2). After conjugation, GMMA were purified with Amicon to remove un-reacted dye and quantified through micro-bicinchoninic acid (μBCA) analysis, and fluorescence exhibition was assessed through HPLC-SEC analysis (Ex/Em = 494/517 nm).

### 4.4. hPBMC Treatments

Following the thawing process, 1 × 10^6^ viable cells per well were seeded in round-bottom, 96-well plates provided by Corning. Subsequently, antigenic stimulation was initiated in the following manner: wild-type *S. sonnei* GMMA and 1790 *S. sonnei* GMMA [27] were utilized at a concentration of 1 μg/mL (protein-based) in flow cytometry experiments to detect intracellular cytokines. The negative control consisted of the use of complete medium (MED). Incubation was carried out for 22 h at 37 °C in an environment with 5% CO_2_.

For flow cytometry analysis involving surface detection, hPBMCs were additionally stimulated with *S. sonnei* GMMA (both wild-type and 1790-GMMA) conjugated with Alexa Fluor-488 [27] at a concentration of 1 μg/mL (protein-based). This incubation step was performed for a duration of 1 h at 37 °C in a 5% CO_2_ atmosphere.

### 4.5. hPBMC Flow Cytometry for Phenotypic Characterization and Intracellular Cytokine Determination

After the various treatments, hPBMCs were subjected to the following procedures. First, a Live/Dead Fixable Near-IR Dead Cell Stain Kit from Invitrogen (Carlsbad, CA, USA) (Catalog Number L10119) was employed to stain the cells. This staining was carried out in the dark at room temperature (RT) for 20 min. Subsequently, the cells were washed twice with PBS and incubated with 2% rabbit serum in PBS for an additional 20 min at RT.

For phenotypic characterization of cell subsets, the cells were stained with a panel of monoclonal antibodies (mAbs), including anti-CD3 (BUV805), anti-CD11c (BV510), anti-HLA-DR (BUV395), anti-CD4 (BV605), anti-CD8 (PerCP-Cy5.5), anti-CD14 (BV786), anti-CD16 (BV421), anti-CD19 (PE-Cy5), anti-CD56 (BV650), anti-CD123 (PE-CF594), anti-CD1c (APC), and anti-TCRγδ (PE-Cy7). The manufacturer’s recommended working dilutions were followed for all mAbs. Incubation with these mAbs took place at RT for 20 min, after which the cells were washed twice with PBS. Subsequently, they were fixed with Cytofix from BD Bioscience (San Jose, CA, USA) for 15 min at +4 °C and then washed twice with PBS. The cells were finally resuspended in 130 μL of PBS containing 2.5 mM of EDTA. Particularly, monocytes were identified as CD3−CD19−CD56−HLADR+CD14+CD16+/− dendritic cells as CD3−CD19−CD56−CD14−HLADR−CD11c+CD123− (mDCs) or CD3−CD19−CD56−CD14−HLADR−CD11c−CD123+ (pDCs), NK cells as CD3−CD19−CD56+CD16+/−, B cells as CD3−CD19+, and T cells as CD3+CD8+ (CD8 T cells), CD3+CD4+ (CD4 T cells), and CD3+CD4−CD8− (γδ T cells).

For intracellular cytokine determination, Brefeldin A (BfA) (5 mg/mL, BD GolgiPlug, San Jose, CA, USA) was added to the cells after 30 min of incubation with the stimuli. The cells were further incubated for a total of 22 h at 37 °C with 5% CO_2_. After this incubation period, the cells were stained for surface markers using anti-CD11c (BV510), anti-HLA-DR (BUV737), anti-CD14 (BV786), anti-CD16 (BV421), anti-CD19 (PE-Cy5), anti-CD56 (BV650), and anti-CD123 (PE-CF594) antibodies from BD Biosciences and anti-TCRγδ (PE-Cy7) antibody from BioLegend (San Diego, CA, USA). The incubation time for these surface marker antibodies was 20 min at RT. Subsequently, the cells were washed twice with PBS and permeabilized with CytoFix/CytoPerm (BD Bioscience) at +4 °C for 20 min. Following two washes with PermWash (BD Bioscience), the cells were treated with 2% rabbit serum in PBS for 20 min at +4 °C to prevent nonspecific binding of antibodies within the cells. Finally, the cells were stained with anti-CD8α (APCR700), anti-CD4 (BV605), anti-CD3 (BUV805), anti-TNFα (BUV395), anti-IFNα (APC), anti-MIP1α (PE), and anti-IFNγ (BV711) antibodies from BD Biosciences and anti-IL6 (PerCP-Cy5.5) antibody from BioLegend. The staining duration for these intracellular markers was 30 min at RT. After staining, the cells were washed twice with PermWash (BD Bioscience). Finally, samples were washed twice with PBS and resuspended in 150 μL of PBS containing 2.5 mM of EDTA.

Flow cytometric analysis was conducted using the BD LSRFortessa X-20 Cell Analyzer, with instrument optimization following the procedure outlined by Perfetto et al. (2006) [53]. Data analysis was performed using FlowJo 10 software from Becton, Dickinson and Company (Franklin Lakes, NJ, USA).

### 4.6. Confocal Microscopy Analysis of GMMA Localization in Monocytes

To examine the localization of GMMA in monocytes, 2 × 10^5^ live, isolated monocytes were seeded in 100 μL per well in round-bottom, 96-well plates provided by Corning. These monocytes were then stimulated with 1 μg/mL of *S. sonnei*-GMMA wild-type and 1790-GMMA conjugated with Alexa Fluor 647. This stimulation occurred for a duration of 1 h at 37 °C in an atmosphere containing 5% CO_2_. As a negative control, complete medium (MED) was used.

Following the stimulation, the cells underwent a series of steps: they were washed once with PBS; they were incubated with 4% formaldehyde for 30 min at RT; and then they were washed once more with PBS. Subsequently, the cells were incubated with anti-GMMA 1790 *S. sonnei* monoclonal antibody and diluted in 1% BSA for 1 h at RT. After incubation, the cells were washed once again with PBS and then incubated with a secondary antibody, Alexa Fluor 546 goat anti-mouse IgG (H+L) from Invitrogen (Catalog Number A11004), which was diluted in 1% BSA, for 30 min at RT in the dark. Ten minutes before the end of this incubation period, a mixture of DAPI (1:5000, Thermo Scientific, Catalog Number 62248) and Cell Mask Deep Red Plasma Membrane (1:1000, Invitrogen, Catalog Number C10046) was added to each well. Following this step, the cells were washed once more and resuspended in PBS. Fixed cells were then transferred onto polylysine-coated plates (Greiner bio-one, Catalog Number 655090, Monroe, NC, USA), and images were acquired using the Opera Phenix instrument from PerkinElmer (Shelton, CT, USA).

### 4.7. RNA Isolation and RNA Sequencing

Monocytes were extracted from cryopreserved hPBMCs originating from six distinct, healthy donors from Tivoli Hospital. Then, 1 × 10^6^ viable monocytes were seeded per well into round-bottom, 96-well plates manufactured by Corning. Subsequently, *S. sonnei* wild-type and 1790-GMMA were applied at concentrations of 0.1 μg/mL and 0.01 μg/mL (protein-based), respectively. As a negative control, MED was employed. Incubation was carried out for a duration of 3 h at 37 °C in an environment with 5% CO_2_. Following this incubation period, the total RNA was purified using the Zymo Direct-zol kit (Direct-zol RNA Miniprep Plus, Zymo Research, Irvine, CA, USA) following the manufacturer’s prescribed procedures. Subsequently, the Agilent RNA 6000 Pico kit (Agilent Technologies, Santa Clara, CA, USA) was utilized to assess the RNA integrity and concentration for each sample. Only samples with an RNA Integrity Number (RIN) of ≥7 and a 260/280 ratio between 1.8 and 2 were considered suitable for further processing.

The generation of cDNA libraries was carried out using 10 ng of RNA per sample with the Revelo RNA-Seq High Sensitivity library preparation kit from Tecan, following the manufacturer’s instructions. The quantity and quality of each resulting library were assessed using the Agilent High Sensitivity DNA Kit Guide from Agilent Technologies, following the manufacturer’s guidelines. Libraries were normalized to create a single equimolar pool with a concentration of 1.5 nM. This pool was subsequently loaded onto the NovaSeq 6000 instrument, following the standard workflow, where the single pool is automatically distributed across all lanes of the flow cell. All essential procedures were executed in accordance with the NovaSeq 6000 Sequencing System Guide [54].

### 4.8. Transcriptomic Analysis

The EdgeR v3.36 Bioconductor package [55] was employed for differential gene expression analysis. Initially, the list of 58,884 annotated genes was filtered by excluding genes with no mapped reads or those with low read counts, resulting in a working dataset of 17,297 genes. Data normalization and dispersion were then calculated using the calcNormFactors and estimateDisp functions, respectively. Finally, we contrasted scaled transcript abundance values between the 1790-GMMA/wild-type GMMA and control samples to identify significantly modulated genes. Genes with an absolute log2 fold change value of ≥1 and a Benjamini–Hochberg adjusted *p*-value (FDR) of ≤0.05 were considered significantly modulated. The DEGs were subsequently subjected to Reactome pathway enrichment analysis using the hypergeometric test, as implemented in the ReactomePA Bioconductor package. Canonical pathways with an enrichment *p*-value of ≤0.05 and FDR of ≤0.2 were regarded as significantly enriched for DEGs.

Additionally, DEGs were mapped into blood transcriptional modules (BTMs) as described by Banchereau R. et al. [33]. Modules containing fewer than 10 mapped genes and those with less than 25% DEGs were excluded, resulting in a final set of 43 BTMs. BTM response scores were computed by determining the percentages of genes that were significantly upregulated or downregulated. If the majority of genes within a module were upregulated, it was assumed to be positively modulated; conversely, if the majority of genes within a module were downregulated, it was considered downregulated.

Finally, the impact of 1790-GMMA and wild-type GMMA treatments on activating a transcriptional biomarker associated with symptomatic H3N2 influenza infection was assessed [33]. Firstly, the response of each gene belonging to the biomarker across samples was normalized by computing z-scores. Subsequently, samples were clustered using hierarchical clustering, as implemented in the Pheatmap R package. Additionally, the molecular distance to the median (MDTM) measure was calculated for each sample by summing the absolute Log2 deviations from the control for each gene in the biomarker. MDTM responses were then compared across treatments through the Kruskal–Wallis test, followed by pairwise comparisons using the Wilcoxon rank sum exact test. *p*-values were corrected for multiple testing using the Benjamini–Hochberg methodology.

### 4.9. Statistical Analysis

To facilitate comparisons among results obtained from stimulated and unstimulated (control) samples, as well as among different stimuli, non-parametric statistical tests were employed. Specifically, the Mann–Whitney test [56] was utilized to assess cytokine production across various experimental conditions. To control for the family-wise error rate, *p*-values were adjusted using the Benjamini–Hochberg method [57]. Pairwise similarity between cytokine profiles was determined using Spearman’s rank correlation coefficient [58].

For comparisons involving flow cytometry data, either the Mann–Whitney test or the paired Wilcoxon signed-rank test [58] was applied. All statistical analyses were conducted in a two-sided manner and were carried out using either GraphPad Prism version 8.0.224 or the SciPy Python library version 1.5.2 [59].

## Data Availability

The data presented in this study are available upon reasonable request from the corresponding author.

## Supplementary Materials

The supporting information can be downloaded at: https://www.mdpi.com/article/10.3390/ijms25021116/s1.
