# Supplementary material for: Molecular Signature of Monocytes Shaped by the Shigella sonnei 1790-Generalized Modules for Membrane Antigens Vaccine
Source: Int J Mol Sci. 2024 Jan 17;25(2):1116. doi: 10.3390/ijms25021116 (PMC10816432; doi:10.3390/ijms25021116)
Supplement: Supplementary file 1 [file ijms-25-01116-s001.zip › ijms-2747928-supplementary.pdf]

Supplementary Figures

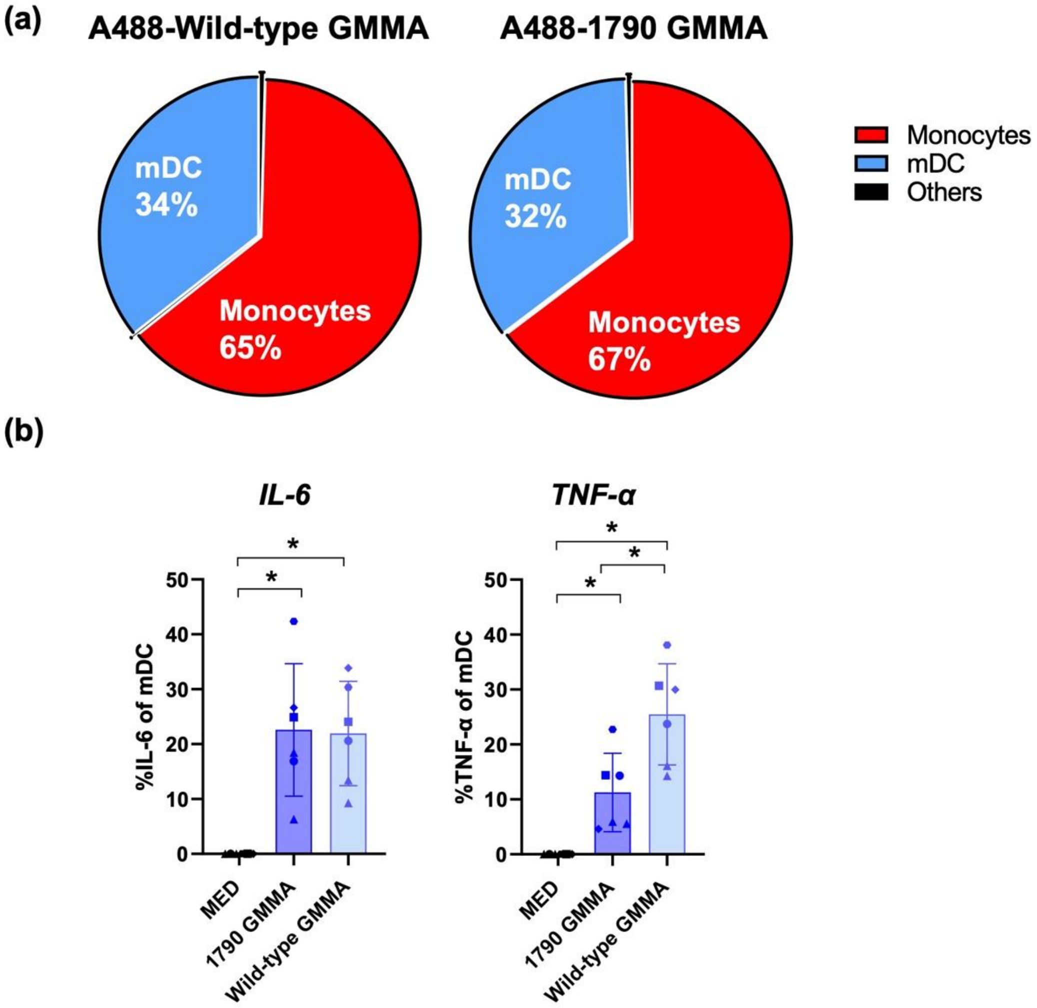

Supplementary Figure S1. (**a**) Flow cytometry analysis for detection of *S. sonnei* A488-Wild-type GMMA and A488-1790 GMMA positive cells after 1 hour of stimulation. Pie chart shows that two cell populations, monocytes (red) and mDC (blue), have the highest percentage of cells positive for both A488-GMMA. The remaining 1% is represented by other immune cell populations (black). (**b**) Intracellular cytokine production in mDC after 22 hours of stimulation with 1790 GMMA (dark blue) and wild-type GMMA (light blue), and Brefeldin A after 30 minutes of treatment. Histograms show a high percentage of IL-6 and TNF-α production by mDC. Percentages were calculated on manually gated cell population. Different dots represent different donors tested (six). Error bars indicate standard deviations (SD). MED is referred to negative control samples (not treated). Significance was estimated using the paired Wilcoxon test (*$p < 0.05$). Significant differences among stimuli are shown explicitly while non-significant differences are not shown.

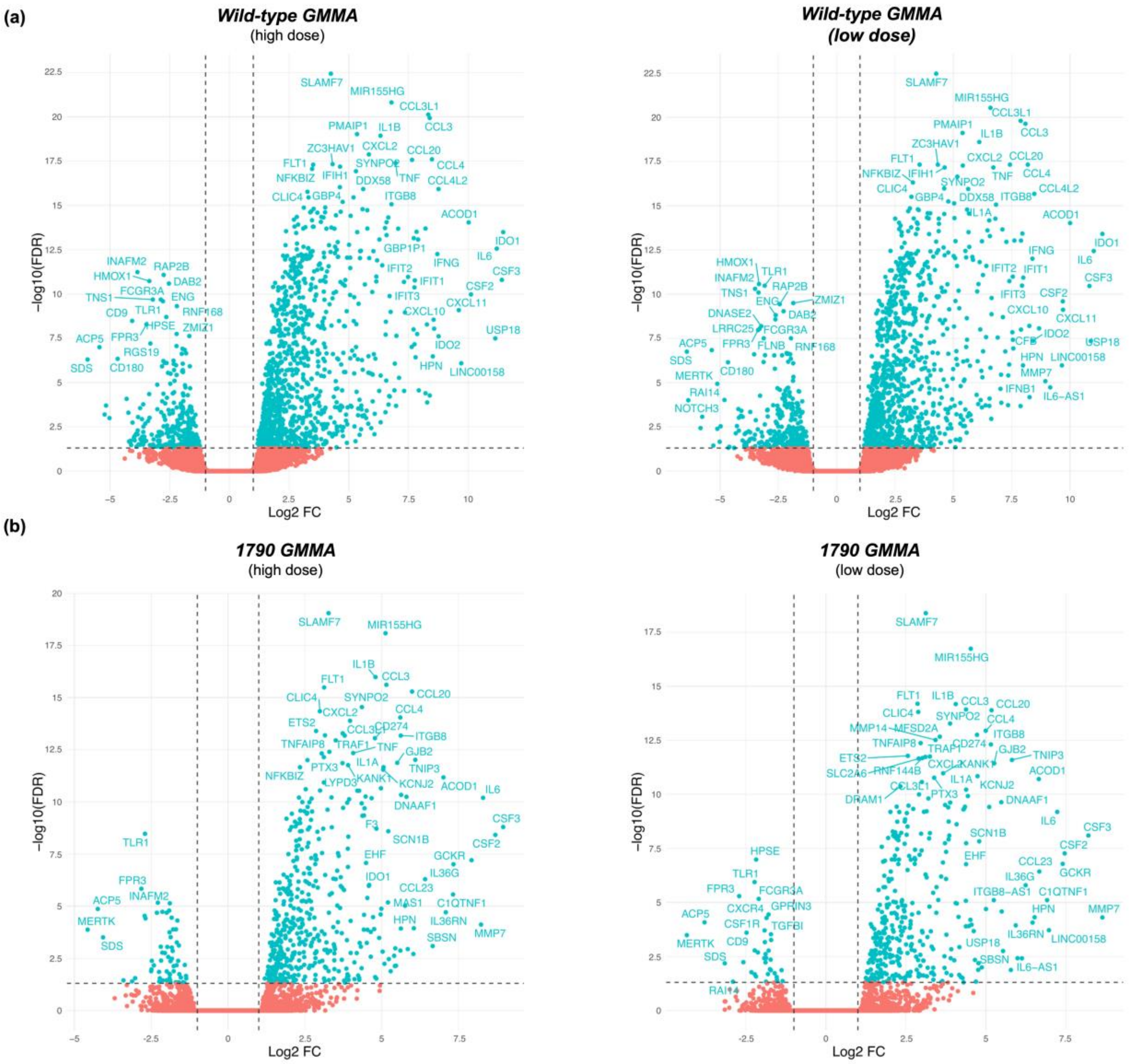

Supplementary Figure S2. Volcano plots representing the transcriptome modulation induced in monocytes upon stimulation with wild-type GMMA (**a**) and 1790 GMMA (**b**) at higher concentration (0,1 μg/ml) and lower concentration (0,01 μg/ml). X axes represent the log2 scaled differential expression compared with the control group, while Y axes represent the statistical significance of the modulation [-log10 scaled Benjamini-Hochberg false discovery rate (FDR)]. Genes with an FDR ≤ 0.05 and |log (FC)| ≥ 1 were assumed to be significantly modulated. Modulated genes are shown as blue dots, up-regulated on the right side and down-regulated on the left side, while not regulated genes are shown as red dots. Vertical dotted lines indicate the +1 and -1 log2 fold change, while the horizontal dotted line indicates the 5% false discovery rate.
